# Band Engineering versus Catalysis: Enhancing the Self-Propulsion of Light-Powered MXene-Derived Metal–TiO_2_ Micromotors To Degrade Polymer Chains

**DOI:** 10.1021/acsami.3c13470

**Published:** 2023-12-22

**Authors:** Mario Urso, Luca Bruno, Sandro Dattilo, Sabrina C. Carroccio, Salvo Mirabella

**Affiliations:** † Dipartimento di Fisica e Astronomia “Ettore Majorana”, Università degli Studi di Catania, via S. Sofia 64, Catania 95123, Italy; ‡ CNR-IMM, via S. Sofia 64, Catania 95123, Italy; § CNR-IPCB, Catania Unit, via Paolo Gaifami 18, Catania 95126, Italy

**Keywords:** MXenes, titanium dioxide, microrobots, Schottky junctions, photocatalysis, water purification, polymers, plastics

## Abstract

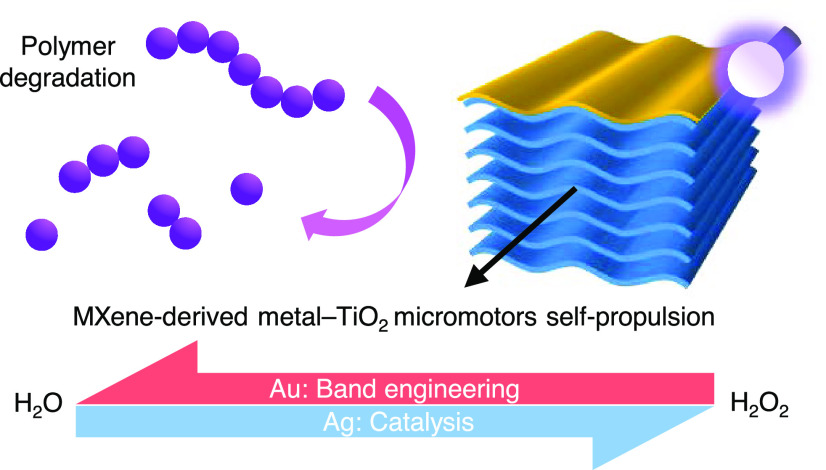

Light-powered micro- and nanomotors based on photocatalytic semiconductors convert light into mechanical energy, allowing self-propulsion and various functions. Despite recent progress, the ongoing quest to enhance their speed remains crucial, as it holds the potential for further accelerating mass transfer-limited chemical reactions and physical processes. This study focuses on multilayered MXene-derived metal–TiO_2_ micromotors with different metal materials to investigate the impact of electronic properties of the metal–semiconductor junction, such as energy band bending and built-in electric field, on self-propulsion. By asymmetrically depositing Au or Ag layers on thermally annealed Ti_3_C_2_T_*x*_ MXene microparticles using sputtering, Janus structures are formed with Schottky junctions at the metal–semiconductor interface. Under UV light irradiation, Au–TiO_2_ micromotors show higher self-propulsion velocities due to the stronger built-in electric field, enabling efficient photogenerated charge carrier separation within the semiconductor and higher hole accumulation beneath the Au layer. On the contrary, in 0.1 wt % H_2_O_2_, Ag–TiO_2_ micromotors reach higher velocities both in the presence and absence of UV light irradiation, owing to the superior catalytic properties of Ag in H_2_O_2_ decomposition. Due to the widespread use of plastics and polymers, and the consequent occurrence of nano/microplastics and polymeric waste in water, Au–TiO_2_ micromotors were applied in water remediation to break down polyethylene glycol (PEG) chains, which were used as a model for polymeric pollutants in water. These findings reveal the interplay between electronic properties and catalytic activity in metal–semiconductor junctions, offering insights into the future design of powerful light-driven micro- and nanomotors with promising implications for water treatment and photocatalysis applications.

## Introduction

Micro- and nanomotors have emerged as cutting-edge technologies in materials science. These miniature devices are capable of navigating through complex environments and performing specific tasks at small scales, promising a wide range of applications and advancements in various fields, such as targeted drug delivery and therapy,^1−4^ environmental monitoring and remediation,^5−7^ biosensing,^8^ and object manipulation.^9,10^

The propulsion mechanisms of micro- and nanomotors are diverse, ranging from chemical reactions, acoustic waves, and magnetic fields to electric fields and light.^11−15^ Among them, light-driven micro- and nanomotors have attracted considerable attention.^16−18^ Light-powered micro- and nanomotors convert light energy into mechanical motion through various mechanisms involving light-absorbing materials on the motor’s surface. When exposed to light, these materials generate a product gradient (neutral or charged) or localized heating, resulting in a concentration, electric potential, or thermal gradient that propels motors through the surrounding fluid by self-diffusiophoresis, self-electrophoresis, or self-thermophoresis.^19^ Common choices for photoactive materials include photocatalytic semiconductor micro- and nanoparticles, such as the UV light-activated TiO_2_ and ZnO,^20,21^ or visible light-activated α-Fe_2_O_3_ and Cu_2_O, prepared by low-cost chemical syntheses.^22,23^ In most cases, these semiconductors are highly symmetric or are limited by the recombination of photogenerated charge carriers. To solve these problems and unlock the self-propulsion ability, thin layers of noble metal catalysts, like Pt, Au, and Ag, are usually deposited through physical vapor deposition methods on the semiconductors’ surface to construct “two-faced” Janus structures, breaking the micro- and nanoparticles’ symmetry and improving charge carrier separation.^24^

Despite the numerous advantages of light-driven metal–semiconductor Janus micro- and nanomotors, several challenges remain. A significant focus has been placed on increasing the velocity of these motors, as it would enable them to accelerate mass transfer-limited chemical reactions or physical processes. One of the initial strategies involved determining the optimal metal to pair with a fixed semiconductor, aiming to enhance the velocity of the micromotor. It was revealed that Pt is the best choice for TiO_2_ micromotors regardless of potential secondary pollution caused by Pt corrosion in water. At the same time, Au has been identified as the optimal choice for BiOI micromotors.^25,26^ Additional studies have shown that using bimetallic coatings or depositing metal layers successively can improve the velocity of photocatalytic micromotors.^27,28^ In addition, increasing metal deposition time, and so metal layer thickness and compactness, has proved to speed up micromotors’ self-propulsion.^29^ This higher velocity is often linked to the larger electrochemical potential difference between the metal and semiconductor. However, an important aspect that remains relatively unexplored is the influence of the electronic properties of the metal–semiconductor junction on the velocity of light-powered micro- and nanomotors. For instance, the interaction between a metal and a semiconductor is known to dramatically alter the behavior of the junction, inducing a semiconductor energy band bending which controls the flow of current from and to the metal.^30^ Understanding and thoroughly investigating this aspect could provide valuable insights into further optimizing the performance of these motors.

MXenes represent a class of 2D materials with the general formula M_*n*+1_X_*n*_T_*x*_ (*n* = 1, 2, 3), where M stands for early transition metals like Ti, Mo, or V, X represents C and/or N, and T_*x*_ denotes the surface-terminating functionality, which can be −O, −F, or −OH.^31,32^ Due to their multilayered structure, resembling an accordion, and high surface area, exfoliated MXenes have gathered significant interest in fabricating innovative and versatile light-driven micromotors. A recent study demonstrated that Pt–Ti_3_C_2_ nanoflakes, derived by ultrasonication-induced delamination of exfoliated MXene microparticles followed by Pt layer deposition, autonomously moved under UV light irradiation in pure water because of the spontaneous formation of superficial TiO_2_.^33^ Another investigation aimed at converting Ti_3_C_2_T_*x*_ MXene microparticles into photocatalytic TiO_2_ through thermal annealing processes that preserved the characteristic multilayered structure of MXenes. After Pt layer deposition and surface decoration with magnetic γ-Fe_2_O_3_ nanoparticles, the resulting micromotors showed self-propulsion in the 3D space under UV light irradiation in pure water thanks to a powerful driving force in the upward direction, characterized by high velocities up to 16 μm s^–1^.^34^ Similarly, another report introduced micromotors based on V_2_C MXene microparticles, Bi nanoparticles, serving as cocatalysts, and magnetic γ-Fe_2_O_3_ nanoparticles. Under visible light irradiation, these micromotors exhibited velocities ranging from approximately 1 μm s^–1^ in pure water to 3 μm s^–1^ in the presence of a high concentration of 5 wt % H_2_O_2_ fuel.^35^ Still, the reliance on Pt or toxic H_2_O_2_ restricts the concrete applicability of these micromachines.

**Scheme 1:**
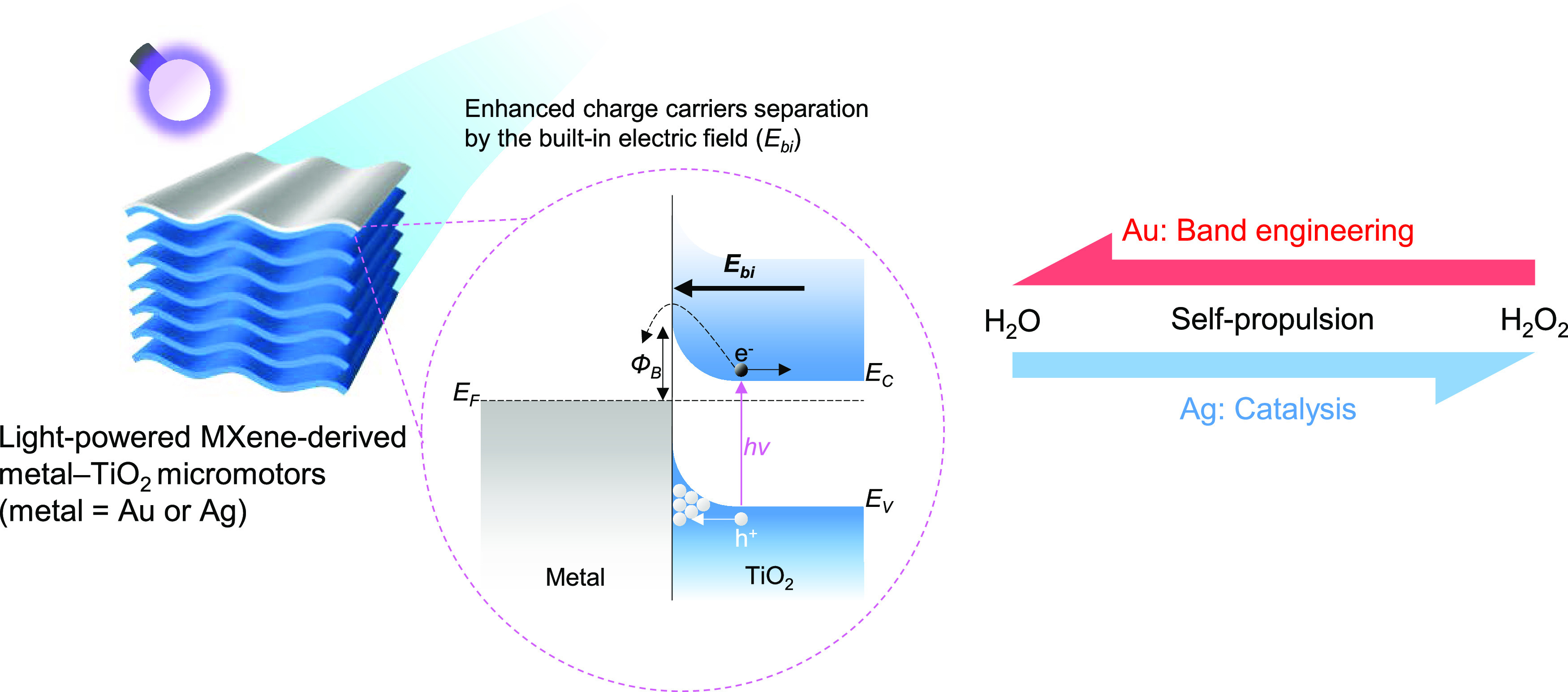
Light-Powered Metal–Semiconductor Micromotors Are Designed by Asymmetrically Depositing Au or Ag Layers on the Surface of Ti_3_C_2_T_*x*_ MXene-Derived TiO_2_ Microparticles These micromotors, characterized by different metal–TiO_2_ interfaces, allow the investigation of the interplay between the effects related to the built-in electric field of the Schottky junction and the metal catalytic properties in obtaining higher self-propulsion velocities in pure water and H_2_O_2_ solutions. *E*_F_, Fermi level; *E*_C_ and *E*_V_, TiO_2_ conduction and valence band energy levels; *h*ν, photon energy; Φ_B_, Schottky barrier; *E*_bi_, built-in electric field; e^–^, electron; and h^+^, hole.

The present study investigates the fabrication, characterization, and light-driven self-propulsion of metal–semiconductor micromotors with different types of metal materials and TiO_2_ as the semiconducting material, aiming to get more insights into how the electronic properties of the metal–semiconductor junction affect their motion behaviors and velocities (Scheme 1). The fabrication of the micromotors involves the thermal annealing process of exfoliated Ti_3_C_2_T_*x*_ MXene microparticles to produce multilayered TiO_2_ microparticles, followed by the asymmetric deposition of Au or Ag layers by the sputtering technique to obtain Janus structures. As confirmed by numerical simulations, Au and Ag’s depositions lead to different bending of TiO_2_ energy bands, forming Schottky contacts characterized by intense electric fields at the metal–semiconductor interface. In pure water, the Schottky junction effect is more significant, with Au–TiO_2_ micromotors showing higher self-propulsion velocities than Ag–TiO_2_ micromotors under UV light irradiation due to the stronger built-in electric field, which efficiently separated photogenerated electron–hole pairs within the semiconductor. This phenomenon also results in hole accumulation beneath the metal surface, favoring the self-electrophoretic mechanism. On the opposite, the introduction of a small amount of 0.1 wt % H_2_O_2_ fuel completely changes the dynamics. The superior catalytic properties of Ag in decomposing H_2_O_2_ give rise to a large product concentration gradient, allowing Ag–TiO_2_ micromotors to overcome Brownian motion and achieve active motion by a self-diffusiophoretic mechanism even in the absence of light. Under UV light irradiation, Ag–TiO_2_ micromotors present a 2-fold increase in velocity than Au–TiO_2_ micromotors due to the synergy between Ag catalytic activity and self-electrophoresis. Finally, the Au–TiO_2_ micromotors were applied in water purification, demonstrating the ability to break down polyethylene glycol (PEG) chains under UV light irradiation in both pure water and H_2_O_2_. These findings shed light on the interplay between electronic properties and catalytic activity in metal–semiconductor junctions, providing valuable insights for designing more powerful and efficient light-driven micro- and nanomotors in the future and promising implications for water treatment and photocatalysis fields.

## Results and Discussion

### Modeling Metal–TiO_2_ Junctions

Before presenting the results of the fabrication, characterization, and light-powered motion analysis of MXene-derived metal–TiO_2_ Janus micromotors with different types of metal layers, it is essential to provide an introduction explaining the significance of diverse metal–semiconductor junctions in the field of self-propelled micro- and nanomotors. The light-driven self-propulsion of semiconductor-based micro- and nanomotors relies on irradiating a photocatalytic semiconductor micro- or nanoparticle with photons of higher energy than the semiconductor’s energy bandgap. The absorption of these photons promotes electrons from the semiconductor’s valence band to the conduction band, leaving holes in the valence band. Then, for the motor to move, photogenerated charge carriers must migrate to the semiconductor’s surface to react with water. However, the creation of photogenerated electron–hole pairs occurs rapidly, on the order of picoseconds, while their migration to the semiconductor’s surface occurs on a longer time scale, ranging from nanoseconds to microseconds.^36^ Consequently, there is a high recombination probability for charge carriers, resulting in no movement of the semiconductor micro- or nanoparticles under light irradiation.

To address this issue, the asymmetric deposition of a metal layer on the semiconductor’s surface can be employed, designing a metal–semiconductor Janus micro- or nanomotor. Then, the most common self-propulsion mechanism involves the reaction between photogenerated holes left in the semiconductor’s valence band, migrated to the surface, and photogenerated electrons transferred to the metal. These charges cause the oxidation and reduction of water according to the following reactions.^37^
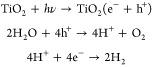
1where TiO_2_ was assumed as the photocatalytic semiconductor material. As a result, the semiconductor side of the micro- or nanomotor acts as a source of protons (H^+^), while the metal side acts as a sink for H^+^. This behavior establishes an H^+^ concentration gradient, which leads to a local electric field around the charged micro- or nanomotor, ultimately inducing its motion by self-electrophoresis.

The work functions of the metal (Φ_m_) and semiconductor (Φ_s_), which represent the minimum energy required to liberate an electron from the materials, play a crucial role in determining the electronic properties of the junction. When Φ_m_ < Φ_s_, an Ohmic contact is obtained, where charge carriers can flow from the metal to the semiconductor and vice versa with low resistance.^30^ On the contrary, when Φ_m_ > Φ_s_, a Schottky contact is obtained, characterized by a rectifying behavior due to the realization of a potential barrier at the interface with the metal governing charge carrier flow. As a consequence, in a Schottky contact, a built-in electric field is generated, which enhances the separation of photogenerated electron–hole pairs in the semiconductor, thereby avoiding the recombination process.

**Figure 1:**
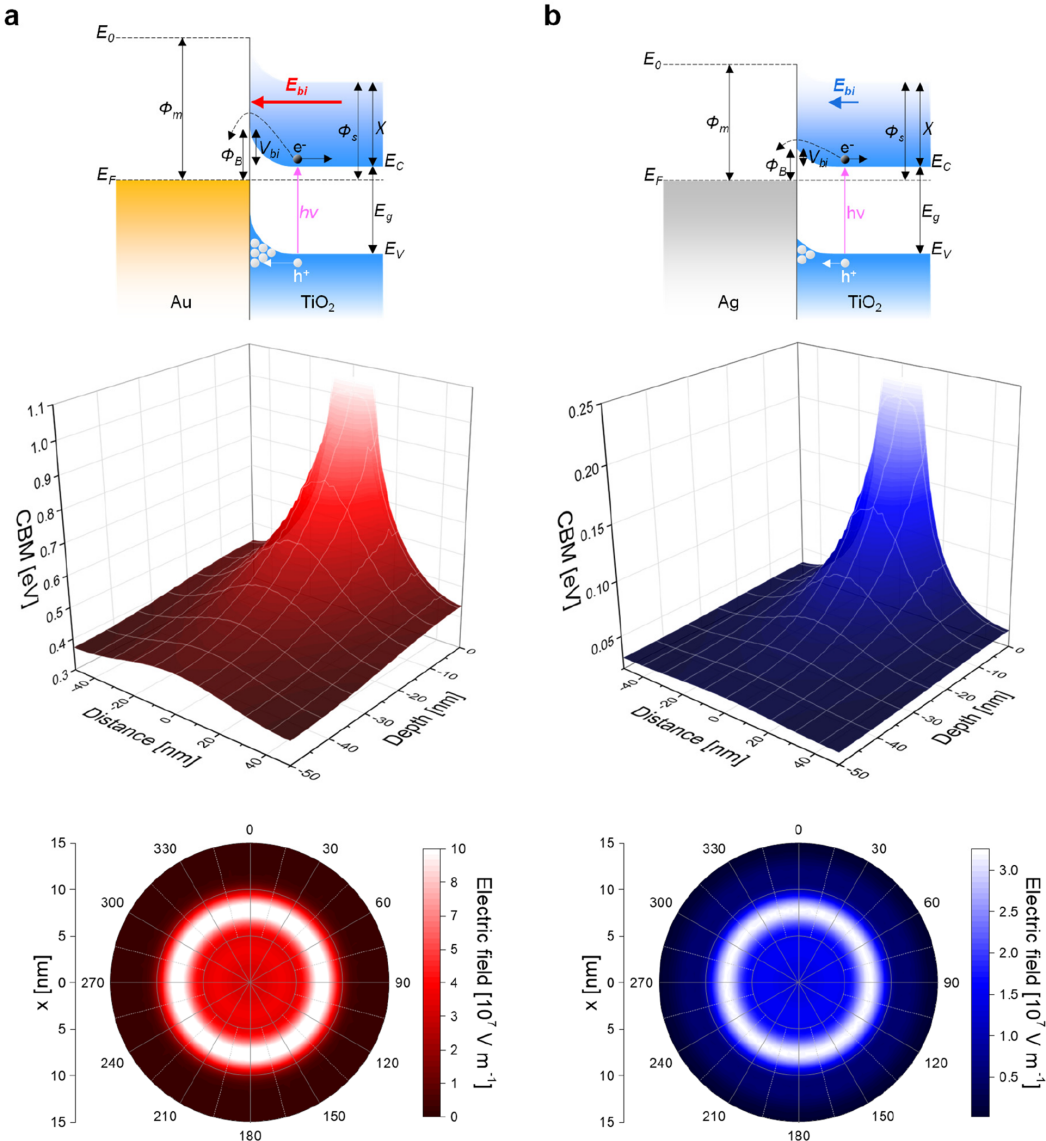
Modeling energy band bending and built-in electric field in metal–TiO_2_ junctions with different types of metal materials. Scheme of the energy band diagram of the metal–TiO_2_ Schottky junction, simulated TiO_2_ conduction band minimum (CBM) energy as a function of depth and distance from the metal nanoparticle’s center, and simulated electric field at the TiO_2_ surface under the metal nanoparticle for (a) Au–TiO_2_ and (b) Ag–TiO_2_ junctions formed by a metal nanoparticle (20 nm in diameter) on the surface of a TiO_2_ microparticle. Φ_m_ and Φ_s_, work functions of the metal and TiO_2_; χ, TiO_2_ electron affinity; *E*_0_, vacuum energy level; *E*_F_, Fermi level; *E*_C_ and *E*_V_, TiO_2_ conduction and valence band energy levels; *E*_g_, TiO_2_ energy bandgap; *h*ν, photon energy; Φ_B_, Schottky barrier; *V*_bi_, built-in potential, *E*_bi_, built-in electric field; e^–^, electron; and h^+^, hole.

Numerical simulations were performed by the Semiconductor Module of COMSOL Multiphysics software to model the energy band banding in semiconductor microparticles after the deposition of layers of different metals. Anatase TiO_2_, an n*-*type semiconductor where electrons are the majority carriers and holes are the minority carriers, was selected as the semiconducting material due to its frequent utilization in the fabrication of light-powered micro- and nanomotors. The chosen metals for this investigation were Au and Ag due to their different work functions (5.47 eV for Au, 4.64 eV for Ag) and their environmental compatibility.^38^ Anatase TiO_2_ relative dielectric permittivity (85),^39^ work function (4.40 eV),^40^ energy bandgap (3.20 eV),^41^ and the mobility^42^ and effective mass^43^ of charge carriers were required for the numerical simulations. No effects related to surface defects or temperature dependence were considered in this model. Nanoscale metal–semiconductor junctions were constructed by placing a single Au or Ag nanoparticle (20 nm in diameter), for simplicity, on the surface of a TiO_2_ microparticle, as illustrated in Figure S1. Au–TiO_2_ and Ag–TiO_2_ contacts behave as Schottky junctions because of the higher work functions of the metals than TiO_2_. In particular, significant upward bending of TiO_2_ conduction and valence bands forms. Consequently, a potential barrier arises *qV*_B_ = *q*Φ_m_*– q*χ for electrons entering TiO_2_ from the metal side, where χ is the semiconductor electron affinity and *q* is the charge. At the same time, the metal–TiO_2_ interface is depleted of electrons and enriched in holes. Electrons in the TiO_2_ conduction band experience a relatively small potential barrier of *qV*_bi_ = *q*Φ_m_*– q*Φ_s_ to transfer to the metal. The energy band diagrams and the simulated maps of the TiO_2_ conduction band minimum (CBM) energy as a function of depth and distance from the metal nanoparticle’s center of Au–TiO_2_ and Ag–TiO_2_ Schottky junctions are compared in Figure 1. The upward bending of the CBM almost approached 1.1 V beneath the Au nanoparticle center and extended 20–30 nm within TiO_2_, while for the Ag nanoparticle, a lower bending was computed (0.2 eV, approximately). This energy band bending generates an electric field under the metal nanoparticles, pointing toward the metals. Figure 1 also reports the simulated 2D maps of the built-in electric field at the TiO_2_ surface below the metal nanoparticles. A 3-fold enhancement in the electric field intensity was calculated for the Au–TiO_2_ Schottky junction (10 × 10^7^ V m^–1^) compared to Ag–TiO_2_ (3 × 10^7^ V m^–1^). Since the electric field is proportional to the spatial derivative of the CBM energy, the highest intensity of the electric fields was found close to the metal nanoparticles’ edges, resulting in distinct “halo” shapes. Such strong and localized built-in electric fields favor the separation of photogenerated electron–hole pairs, avoiding unwanted recombination phenomena and efficiently utilizing absorbed light.

It is reasonable to ask if the built-in electric field intensity significantly influences the motion of light-driven micro- and nanomotors. If so, careful band engineering of metal–semiconductor junctions promises to be the key to achieving powerful light-powered propulsive forces.

### Fabrication and Characterization of MXene-Derived Metal–TiO_2_ Micromotors

The interaction between metal and semiconductor in metal–semiconductor Schottky junctions gives rise to interesting phenomena that control the photogenerated charge carriers’ recombination and flow at the interface. The type and strength of these effects depend on the specific combination of the metal and semiconductor materials used. However, their impact on the light-powered motion of metal–semiconductor-based micro- and nanomotors, if any, has not been exhaustively elucidated yet. In this study, the light-driven self-propulsion of micromotors based on a semiconductor in contact with different metals has been investigated. The semiconductor material chosen for this examination is a Ti_3_C_2_T_*x*_ MXene-derived TiO_2_, combining the high photocatalytic activity of TiO_2_ under UV light irradiation with the accordion-like multilayered structure of exfoliated MXenes, which is highly desirable for practical applications. As for the metals, Au and Ag were selected as the metal materials to realize metal–semiconductor interfaces such as those modeled in Figure 1 and for their biocompatibility compared to Pt, whose corrosion in water potentially causes harm to the environment and health.

**Figure 2:**
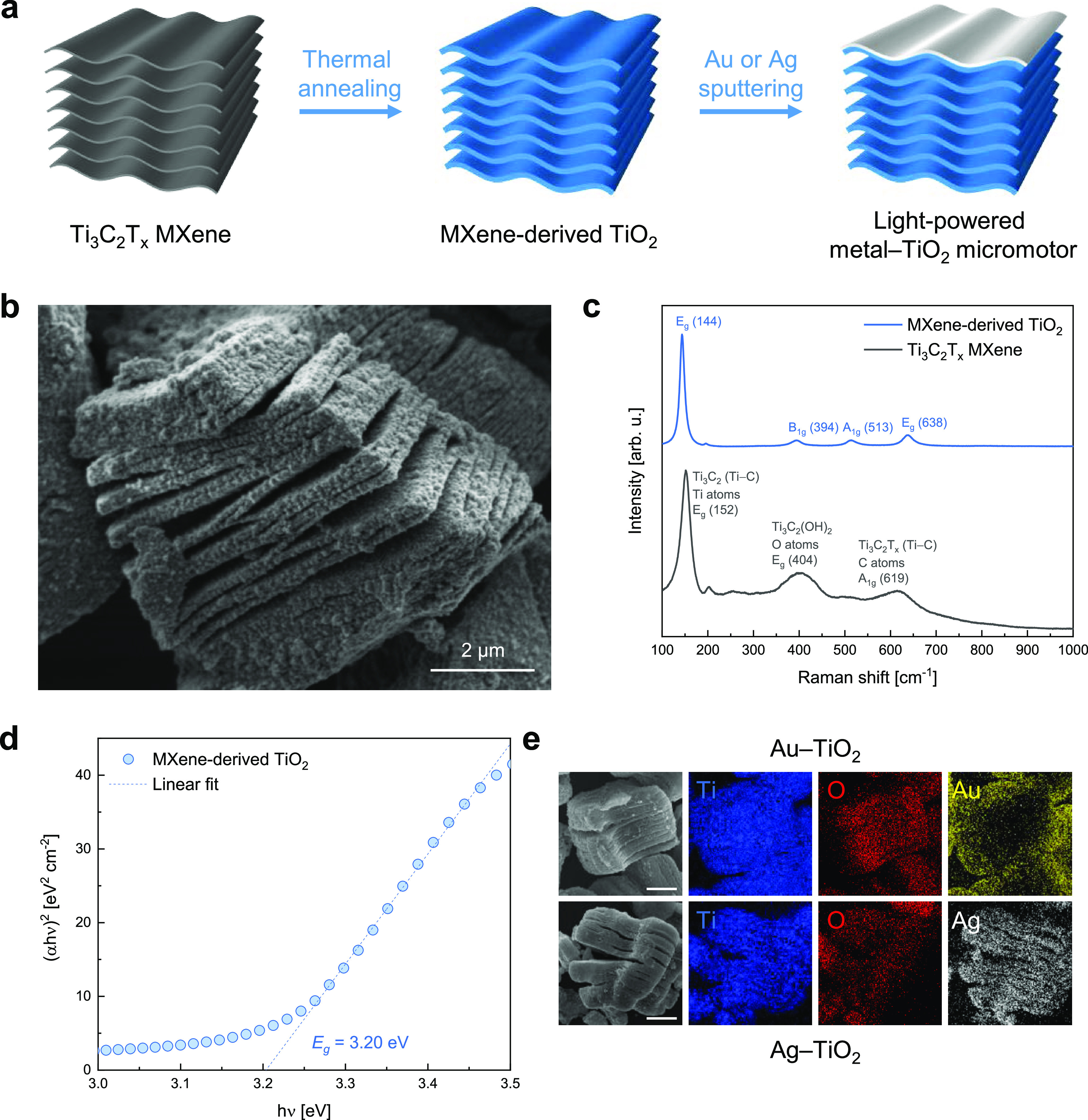
Fabrication and characterization of MXene-derived metal–TiO_2_ micromotors. (a) Scheme of the fabrication steps. (b) SEM image of an MXene-derived TiO_2_ microparticle. (c) Raman spectra of Ti_3_C_2_T_x_ MXene and MXene-derived TiO_2_ microparticles. (d) Tauc plot for bandgap energy (*E*_g_) determination of MXene-derived TiO_2_ microparticles. (e) EDX elemental mapping images for Ti, O, Au, and Ag in MXene-derived metal–TiO_2_ micromotors. Scale bars are 2 μm.

The different fabrication steps of MXene-derived metal–semiconductor micromotors are illustrated in Figure 2a. Exfoliated Ti_3_C_2_T_*x*_ microparticles were thermally annealed at 550 °C for 0 min, i.e., an annealing process where the temperature rump-up is immediately followed by the ramp-down, in synthetic air to induce the oxidation of the Ti_3_C_2_T_*x*_ into TiO_2_. Despite the oxidation of Ti_3_C_2_T_*x*_ to TiO_2_ starting at a lower temperature, previous studies suggested that the thermal annealing process at 550 °C for 0 min results in the optimal morphological, structural, electrochemical, and photocatalytic properties of the obtained TiO_2_.^34,44^ To enable light-powered motion, it was essential to break the symmetry of the semiconductor microparticle. To achieve this, MXene-derived TiO_2_ microparticles were positioned in front of the Au and Ag targets of a sputter coater. This setup allowed for the asymmetric deposition of thin metal layers with a nominal thickness of about 80 nm on the MXene-derived TiO_2_ microparticles’ surface. The scanning electron microscopy (SEM) image shown in Figure 2b provides a closer look at the surface morphology of an MXene-derived TiO_2_ microparticle. In comparison to the pristine MXene (Figure S2), the previously smooth surface of Ti_3_C_2_T_*x*_ multilayer stacks was transformed into aggregated TiO_2_ nanoparticles. Despite this transformation, the characteristic multilayered structure of the Ti_3_C_2_T_*x*_ MXene was maintained due to the rapid thermal annealing process. This preservation of the multilayered structure is especially promising for those applications where contact with the photocatalyst plays a crucial role, such as water purification.

The successful conversion of Ti_3_C_2_T_*x*_ into anatase TiO_2_ was convincingly demonstrated through Raman spectroscopy. In Figure 2c, the Raman spectra of both Ti_3_C_2_T_*x*_ MXene and MXene-derived TiO_2_ microparticles are compared. For Ti_3_C_2_T_*x*_ MXene microparticles, three distinctive bands were observed in the Raman spectrum: the band at 152 cm^–1^ was attributed to in-plane Ti–C vibrations in Ti_3_C_2_ with *E*_g_ symmetry, the band at 404 cm^–1^ was ascribed to the in-plane vibrations of the O atoms in OH-terminated MXene (Ti_3_C_2_(OH)_2_) with *E*_g_ symmetry, and the band at 619 cm^–1^ corresponded to out-of-plane Ti–C vibrations in Ti_3_C_2_ with A_1g_ symmetry, in agreement with a previous study.^45^ On the contrary, the Raman spectrum of MXene-derived TiO_2_ microparticles exhibited the characteristic bands of anatase TiO_2_, including *E*_g_ symmetry band at 144 cm^–1^, B_1g_ symmetry band at 394 cm^–1^, A_1g_ symmetry band at 513 cm^–1^, and *E*_g_ symmetry band at 638 cm^–1^.^46^ These results are coherent with the XRD pattern of the MXene-derived TiO_2_ microparticles prepared by the same experimental procedure and reported in a previous manuscript,^34^ which revealed the anatase crystalline phase of TiO_2_. It is important to note that anatase TiO_2_ is known for the highest photocatalytic efficiency among the TiO_2_ polymorphs, making this transformation highly significant for potential applications in photocatalysis.^47^

The optical properties of MXene-derived TiO_2_ microparticles were investigated using UV–visible spectroscopy. Particularly, the Tauc plot in Figure 2d was derived from the absorbance measurement, and the energy bandgap of MXene-derived TiO_2_ microparticles was determined to be 3.20 eV by extrapolating the linear part of the plot. This value is suitable for the absorption of UV light by the following micromotors. Notably, this value aligns perfectly with the expected optical bandgap for anatase TiO_2_, whose obtainment was also supported by the results of Raman analysis. Furthermore, the observed change in the color of the powders after annealing is consistent with these findings, as they transitioned from the black color of Ti_3_C_2_T_*x*_ MXene to the white color of TiO_2_.

The effective fabrication of metal–semiconductor Janus micromotors was demonstrated through energy-dispersive X-ray (EDX) spectroscopy to obtain elemental mapping images of MXene-derived TiO_2_ microparticles after the metal deposition step (Figure 2e). These images clearly show the presence and spatial distribution of Ti, O, Au, and Ag elements. The uniform distribution of Ti and O elements over the MXene-derived TiO_2_ microparticles confirmed the successful transformation of the MXene precursor into TiO_2_. Additionally, the images provide evidence of the presence of Au and Ag elements after the sputtering process. It is worth noting that these images do not show the characteristic Janus structure which has been extensively observed for spherical microparticles after the deposition of a metal layer by the sputtering technique.^20,25,48^ This is due to the high intrinsic asymmetry, multilayered structure, and rough surface of the MXene-derived TiO_2_ microparticles, which makes it challenging to visualize the boundary between the metal-coated side of the microparticles and the uncoated one. Nonetheless, in the EDX mapping image of the Au–TiO_2_ micromotor, the Au element is more present on the edge of the micromotor, suggesting the achievement of an asymmetric structure that can allow its directional propulsion under UV light irradiation.

### Motion Behavior of MXene-Derived Metal–TiO_2_ Micromotors

**Figure 3:**
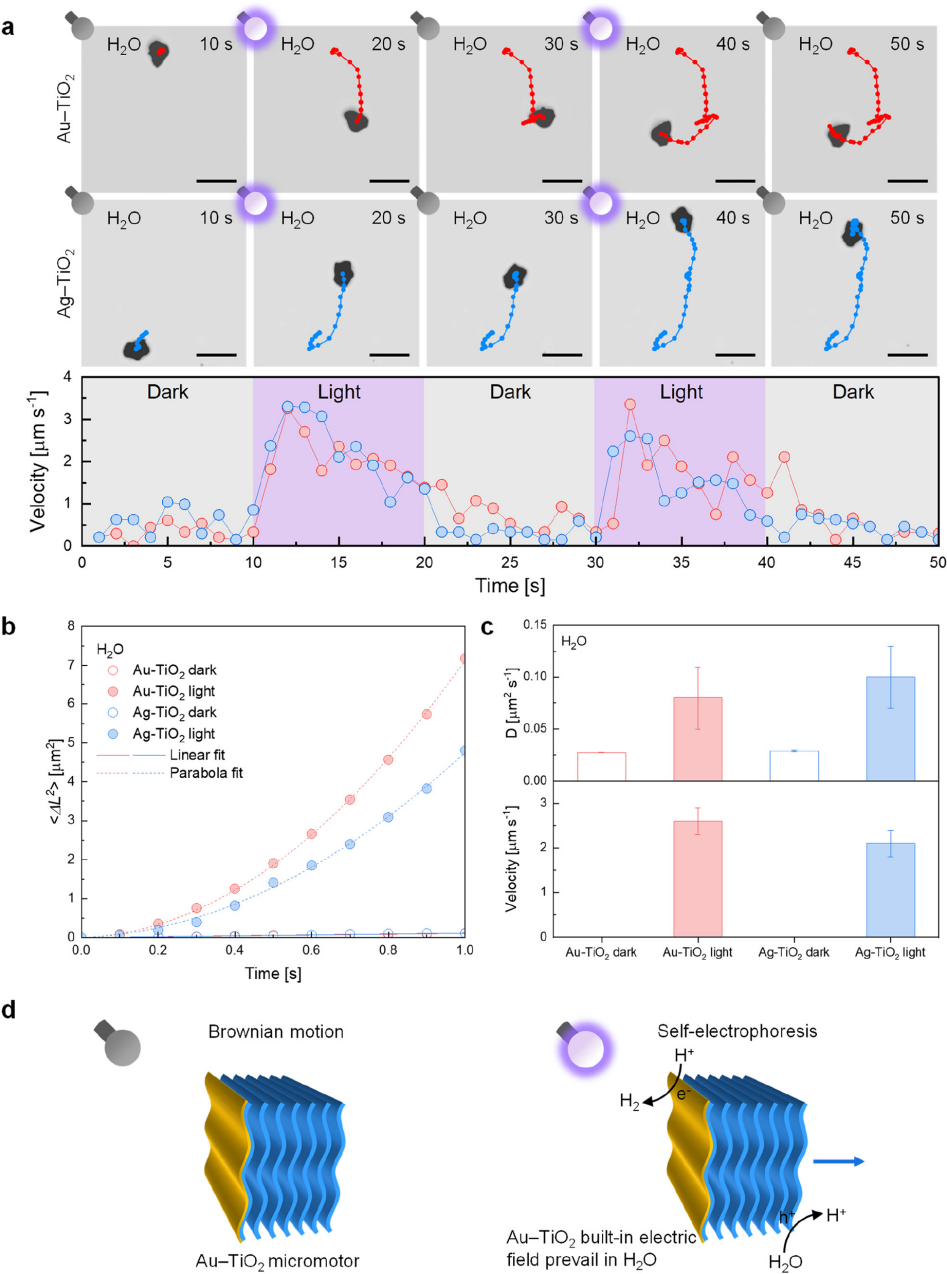
Motion behavior of MXene-derived metal–TiO_2_ micromotors in pure water. (a) Time-lapse micrographs showing the trajectories of an Au–TiO_2_ micromotor and an Ag–TiO_2_ micromotor for successive on–off switching of UV light irradiation at time intervals of approximately 10 s in pure water, and the corresponding instantaneous velocities as a function of time. Scale bars are 5 μm. (b) Mean squared displacement (MSD) data fitting of several metal–TiO_2_ micromotors in the absence (dark) and presence (light) of UV light irradiation in pure water. MSD data were fitted based on eqs 3 and 4. Error bars are not shown for clarity. (c) Average diffusion coefficients and velocities obtained from MSD data fitting. Error bars represent the standard deviation. (d) Scheme of Au–TiO_2_ micromotor motion mechanisms in the absence and presence of UV light irradiation in pure water.

The motion behavior of MXene-derived Au–TiO_2_ and Ag–TiO_2_ micromotors was investigated in pure water to disclose the potential influence of the higher built-in electric field in the Au–TiO_2_ Schottky junction compared to the Ag–TiO_2_ one, originating from the different work functions of the metals. Initially, a control experiment verified that MXene-derived TiO_2_ microparticles show only Brownian motion under UV light irradiation in pure water, as indicated by the representative trajectories in Figure S3. This observation is in agreement with previous studies demonstrating that the self-propulsion of metal-free single-component photocatalytic semiconductor micro- and nanoparticles has been obtained for intrinsically asymmetric structures or upon exposure to directional illumination, eventually in the presence of additional H_2_O_2_ fuel.^49^ The asymmetric deposition of a metal layer was expected to turn MXene-derived TiO_2_ microparticles into efficient, light-powered micromotors. In this regard, the first experiment aimed at evaluating micromotors’ response to repeated on–off switches of the UV light source at time intervals of approximately 10 s in pure water (Movie S1). Figure 3a reports time-lapse micrographs showing the trajectories of an Au–TiO_2_ micromotor and an Ag–TiO_2_ micromotor. Both micromotors exhibited no self-propulsion in the dark in pure water. However, upon turning on UV light irradiation, micromotors manifested the self-propulsion ability, which resulted in a net displacement. Once the dark condition was restored, the micromotors’ movement rapidly stopped. Therefore, in pure water, micromotors could rapidly change their motion status following the presence or absence of UV light. It is worth noting that after the UV light source was turned off, the micromotors occasionally displayed a significant displacement due to recoil phenomena rather than Brownian motion only, as observed after 20 s for the Au–TiO_2_ micromotor in Figure 3a. Nonetheless, the distinct behavior under UV light irradiation was confirmed by the remarkable rise of the instantaneous velocity of the micromotors as a function of time. In the dark, micromotors presented a similar instantaneous velocity of 0–1 μm s^–1^, which increased to 1–3 μm s^–1^ under UV light irradiation.

To get more insights into the nature of the micromotors’ motion behavior in dark and light conditions in pure water, movies of several micromotors were recorded and tracked to obtain their trajectories and calculate the mean squared displacement (MSD), denoted as ⟨Δ*L*^2^⟩ [μm^2^]. The magnitude of ⟨Δ*L*^2^⟩ reflects the strength of the propulsive force, while its variation over time offers insight into the type of motion. For an ensemble of particles at the time interval Δ*t* [s], ⟨Δ*L*^2^⟩ is defined as:
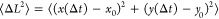
2where *x*(Δ*t*) and *y*(Δ*t*) [μm] are the coordinates of the *i*th particle at the time interval Δ*t*, *x*_0_, and *y*_0_ are the initial coordinates of the *i*th particle, and the brackets “⟨⟩” indicate the average over numerous particles.^50^ For a spherical particle on a plane experiencing Brownian motion, i.e., random fluctuations of the particle’s position due to the diffusion process, ⟨Δ*L*^2^⟩ is linear with Δ*t*:
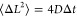
3where *D* [μm^2^ s^–1^] is the diffusion coefficient. In some cases, ⟨Δ*L*^2^⟩ varies as Δ*t*^α^, with α > 1, and the particles’ motion is referred to as “superdiffusive.” For particles in the ballistic motion regime, α = 2, and ⟨Δ*L*^2^⟩ obeys the following relationship:
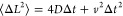
4where *v* [μm s^–1^] is the velocity. This theoretical framework was used to model the MSD data of micromotors (the results of MSD data fitting are reported in Table S1). For both types of micromotors, MSD analysis revealed the linearity between ⟨Δ*L*^2^⟩ and Δ*t* in the dark according to eq 3, which suggested that micromotors displayed Brownian motion with random displacements in the absence of UV light in pure water. Instead, ⟨Δ*L*^2^⟩ followed a quadratic relationship with Δ*t*, as stated by eq 4, under UV light irradiation, demonstrating the self-propulsion of micromotors with directional motion in pure water. This finding is in agreement with the trajectories in the time-lapse micrographs in Figure 3a. Noteworthy, ⟨Δ*L*^2^⟩ values of Au–TiO_2_ were higher than Ag–TiO_2_. Consequently, by averaging on multiple micromotors, it was found that Au–TiO_2_ micromotors’ light-driven motion in pure water was more powerful than that of Ag–TiO_2_ micromotors. Moreover, compared to the previously published TiO_2_@Ti_3_C_2_/Pt micromotors, Au–TiO_2_ micromotors reached a higher ⟨Δ*L*^2^⟩ after 1 s (7 vs 1.5 μm^2^, approximately) under similar experimental conditions.^33^

MSD data fitting allowed us to determine the diffusion coefficients and velocities of micromotors in the absence and presence of UV light irradiation in pure water. In the dark, micromotors had comparable diffusion coefficients (0.027–0.029 μm^2^ s^–1^). Under UV light irradiation, a 3-fold increase in the diffusion coefficients was found (0.08–0.1 μm^2^ s^–1^). Therefore, diffusion coefficients were similar independently to the type of metal material. In contrast, under UV light irradiation, a higher velocity was obtained for Au–TiO_2_ micromotors than Ag–TiO_2_ (2.6 vs 2.1 μm s^–1^), which is explained by the stronger built-in electric field at the Au–TiO_2_ interface. Previous reports, which focused on comparing the velocity of light-driven metal–semiconductor Janus micromotors prepared with different metals, utilized electrochemical measurements to validate velocity results. In this context, metal–semiconductor micromotors were modeled as two electrodes, one for the metal material and the other one for the semiconductor material, with distinct electrochemical potentials.^26,28^ Then, the researchers argued that the larger the potential difference between the two electrodes, the larger the resulting micromotors’ velocity. For example, Maric et al. prepared metal–TiO_2_ micromotors using Pt, Cu, Fe, Ag, and Au.^25^ The electrochemical potential analysis allowed them to justify the higher velocity of the Pt–TiO_2_ micromotors. Nevertheless, for Fe–TiO_2_ and Cu–TiO_2_ micromotors, it predicted a lower velocity than Ag–TiO_2_, in contrast with the motion experiments results. This discrepancy is explained by the fact that even though the electrochemical potential difference generally provides valuable information about the velocity of micromotors, it may not consider condensed matter physics phenomena occurring upon the contact between the metal and the semiconductor materials, such as the establishment of a Schottky junction, which affects the charge carrier transfer process at the metal–semiconductor interface. In fact, Fe and Cu have generally larger work functions (4.81 and 4.94 eV) than Ag (4.74 eV), similar to the case of Au.^38^

The built-in electric field at metal–TiO_2_ Schottky junctions potentially has a double-edged sword effect: on the one hand, it promotes the separation of photogenerated carriers and the accumulation of holes at the interface; on the other hand, the higher potential barrier is detrimental to the electron transfer from the semiconductor to the metal. The investigation of the motion behavior of MXene-derived Au–TiO_2_ and Ag–TiO_2_ micromotors concludes that the stronger built-in electric field at the Au–TiO_2_ interface positively impacts micromotors’ light-driven self-propulsion ability. This result suggests that the higher density of holes beneath the Au layer enhances the reaction rate of the oxidation of water to H^+^, generating a larger concentration gradient of H^+^ and, thus, a more intense local-electric field responsible for micromotors’ self-electrophoresis, as illustrated in Figure 3d. Thereby, in metal–TiO_2_ micromotors, such a positive effect surpasses the negative effect associated with the higher potential barrier for electron transfer.

It is worth noting that the aim of this study was not to achieve velocities higher than those reported in the literature. Nonetheless, Au–TiO_2_ micromotors achieved a higher or comparable velocity than γ-Fe_2_O_3_–Bi–V_2_C micromotors and many other fuel-free metal–semiconductor Janus micromotors tested in similar conditions.^25,26,28,35^ The metal–TiO_2_ micromotors in this study were prepared following the same fabrication procedure of the previously reported MXene-derived γ-Fe_2_O_3_/Pt/TiO_2_ microrobots.^34^ The only difference is the presence of a Pt layer rather than Au or Ag layers and the inclusion of magnetic nanoparticles to provide the microrobots with magnetic properties. Pt is known to be a better catalyst than Au and Ag for H_2_ production from water. Therefore, it is not surprising that the Au–TiO_2_ micromotors have a lower velocity than γ-Fe_2_O_3_/Pt/TiO_2_ microrobots under UV light irradiation in pure water (2.6 vs 16 μm s^–1^). Still, it is worth noting that Au–TiO_2_ micromotors were powered using a 30 times lower intensity of the UV light source than γ-Fe_2_O_3_/Pt/TiO_2_ microrobots (∼50 vs ∼1500 mW cm^–2^). Besides, previous studies suggest that the velocity of Au–TiO_2_ micromotors can be further increased by improving the compactness of the metal layer, for example by prolonging the sputtering deposition or using nonlayered semiconducting microparticles as the main building block.^51,52^

To assess the applicability of micromotors in real scenarios, such as in wastewater purification, the UV light-driven self-propulsion of Au–TiO_2_ and Ag–TiO_2_ micromotors was investigated in raw wastewater, i.e., before entering the wastewater treatment plant and being subjected to any purification process. Figure S4 reports the photograph and micrograph of the wastewater sample, which reveal the massive presence of solid impurities. In this complex environment, the micromotors did not manifest the ability to autonomously move under UV light irradiation, being stuck on the microscope glass slide or obstructed by the surrounding microparticles. Nonetheless, wastewater can be first treated to remove and release the contaminants in a second vessel, where the micromotors can induce their photocatalytic degradation under UV light irradiation. This approach allows for the confinement of potential secondary pollution. Alternatively, the micromotors can be loaded with magnetic nanoparticles, powered by an external magnetic field in wastewater and, simultaneously, activated by UV light irradiation to catch and degrade the pollutants.^7^

Since UV light is not biocompatible, the self-propulsion ability of Au–TiO_2_ and Ag–TiO_2_ micromotors was also investigated under visible light irradiation in pure water. However, the micromotors displayed Brownian motion only (Movie S4). This result is in agreement with the measured energy bandgap of the MXene-derived TiO_2_ microparticles (3.20 eV), which indicates that the micromotors can be activated by UV light only.

**Figure 4:**
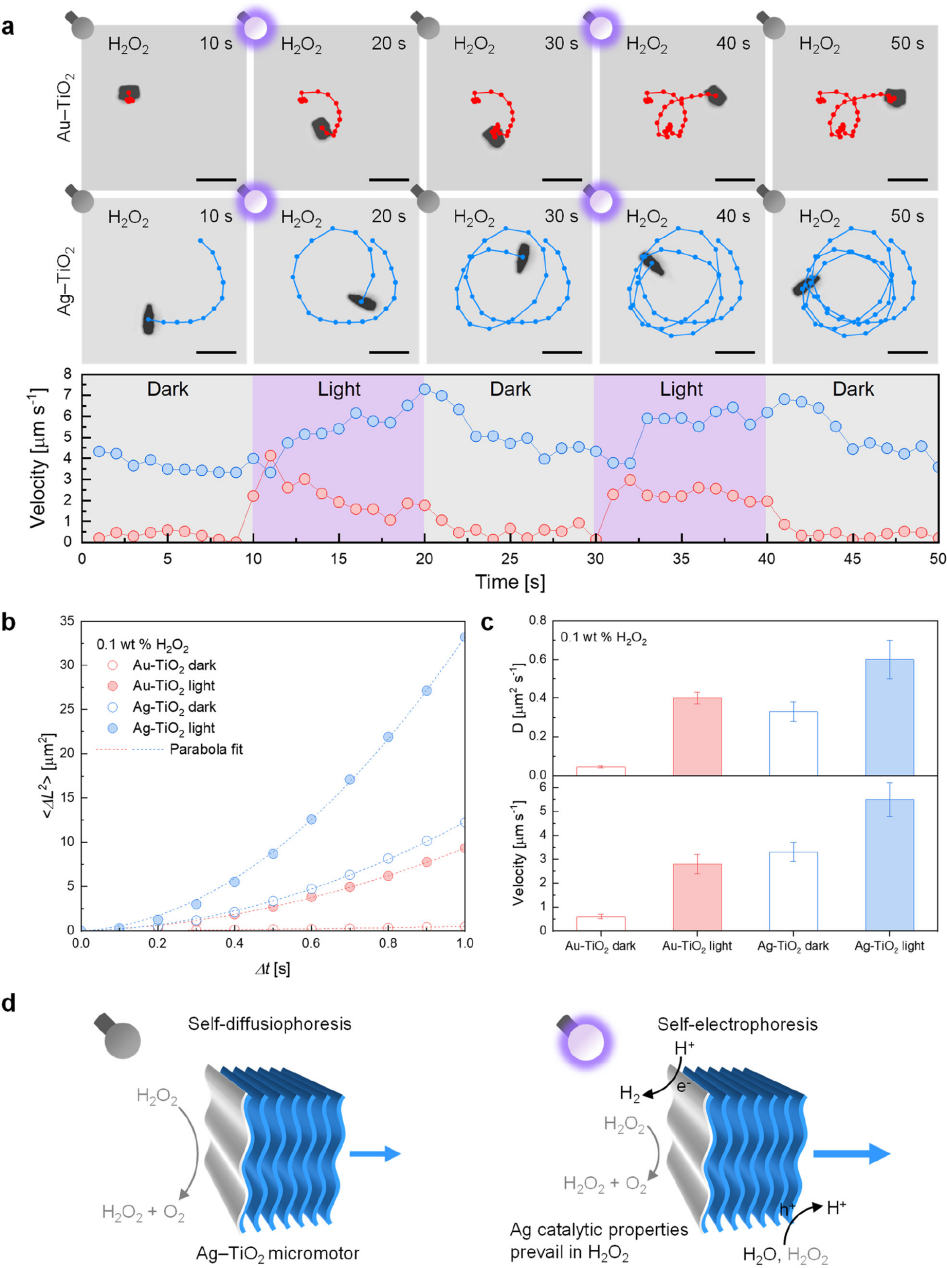
Motion behavior of MXene-derived metal–TiO_2_ micromotors in a low concentration of 0.1 wt % H_2_O_2_. (a) Time-lapse micrographs showing the trajectories of an Au–TiO_2_ micromotor and an Ag–TiO_2_ micromotor for successive on–off switching of UV light irradiation at time intervals of approximately 10 s in 0.1 wt % H_2_O_2_, and the corresponding instantaneous velocities as a function of time. Scale bars are 5 μm. (b) Mean squared displacement (MSD) data fitting of several metal–TiO_2_ micromotors in the absence (dark) and presence (light) of UV light irradiation in 0.1 wt % H_2_O_2_. MSD data were fitted based on eqs 3 and 4. Error bars are not shown for clarity. (c) Average diffusion coefficients and velocities obtained from MSD data fitting. Error bars represent the standard deviation. (d) Scheme of Ag–TiO_2_ micromotors motion mechanisms in the absence and presence of UV light irradiation in 0.1 wt % H_2_O_2_.

While this discussion may seem comprehensive, it must be noted that many light-powered micro- and nanomotors cannot move in pure water and require additional fuels to manifest their self-propulsion ability. Among the fuels, H_2_O_2_ is the most commonly reported, regardless of its potential toxicity at high concentrations. H_2_O_2_ contributes to the micro- and nanomotors’ motion with the following reactions involving photogenerated charge carriers.^37^
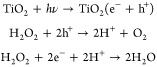
5Therefore, to deepen the comparison and understanding of the performance of different metal–semiconductor junctions and related electronic properties, the motion behavior of MXene-derived Au–TiO_2_ and Ag–TiO_2_ micromotors was also examined at the low concentration of 0.1 wt % H_2_O_2_. Once again, the first experiment explored the response of micromotors in dark and light conditions, influenced by the presence of the fuel (Movie S2). The time-lapse micrographs in Figure 4a indicate no significant difference for the Au–TiO_2_ micromotors in 0.1 wt % H_2_O_2_ compared to pure water: random fluctuations of the micromotor’s position were observed in the dark, and a directional motion was noted under UV light irradiation. Conversely, the Ag–TiO_2_ micromotor presented a completely different scenario, characterized by directional motion in both the presence and absence of UV light irradiation. This behavior was detected for successive on–off switching of the UV light source, during which the micromotor preserved its mobile status. The lack of control over the on–off status of the Ag–TiO_2_ micromotor compared to that of the Au–TiO_2_ micromotor was reflected in the temporal variation of its instantaneous velocity. For the Au–TiO_2_ micromotor, the on–off switching of the UV light source was followed by a rapid rise–decrease of the velocity (from 0–1 μm s^–1^ to 2–3 μm s^–1^). On the other hand, the Ag–TiO_2_ micromotor displayed a high and constant velocity in the dark (3–5 μm s^–1^), which further increased under UV light irradiation (5–7 μm s^–1^). Hence, the reaction between Ag and H_2_O_2_ rendered the micromotor active even in the absence of light, and its activity was amplified upon exposure to UV light irradiation. Of note, the trajectories of Au–TiO_2_ micromotors and Ag–TiO_2_ micromotors in both Figures 3a and 4a are clockwise and anticlockwise, respectively. Even so, it was not possible to control the direction of the motion of micromotors, forcing their movement along a clockwise or anticlockwise rotation.

MSD analysis was employed to unambiguously determine the type of motion of micromotors in 0.1 wt % H_2_O_2_. Fitted MSD data are shown in Figure 4b (the results of MSD data fitting are reported in Table S1). First, a linear fit of MSD data of Au–TiO_2_ micromotors in the dark using eq 3 was attempted. The linearity between ⟨Δ*L*^2^⟩ and Δ*t* was hinted by the absence of a net displacement in the time-lapse images of the Au–TiO_2_ micromotor in the dark in 0.1 wt % H_2_O_2_. Nevertheless, the inconsistency of the fitting results suggested a superdiffusive motion behavior rather than Brownian motion. Then, by assuming a ballistic motion and a quadratic relationship between ⟨Δ*L*^2^⟩ and Δ*t* as in eq 4, a satisfactory fitting was attained. This outcome revealed the underlying reaction between Au and H_2_O_2_, whose contribution was not powerful enough to overcome Brownian motion. Under UV light irradiation, ⟨Δ*L*^2^⟩ of Au–TiO_2_ micromotors followed a parabola, as expected from the observed directional motion in 0.1 wt % H_2_O_2_ in the time-lapse micrographs in Figure 4a. Regarding the Ag–TiO_2_ micromotors in 0.1 wt % H_2_O_2_, the hypothesis of ballistic motion was confirmed by fitting MSD data with eq 4. Notably, MSD data of Ag–TiO_2_ micromotors in the dark were already higher than UV light irradiated Au–TiO_2_ micromotors, before further increasing for UV light-irradiated Ag–TiO_2_ micromotors. This observation highlighted a large difference between the metals originating from the presence of the H_2_O_2_ fuel.

The diffusion coefficients and velocities of micromotors in dark and light conditions in the presence of 0.1 wt % H_2_O_2_ were obtained and are compared in Figure 4c. Under UV light irradiation, the diffusion coefficients increased for both types of micromotors, with Ag–TiO_2_ micromotors showing the highest diffusion coefficient (0.6 μm^2^ s^–1^). Ag–TiO_2_ micromotors exhibited a high diffusion coefficient also in the dark (0.33 μm^2^ s^–1^), which was significantly larger than Au–TiO_2_ micromotors under the same condition (0.045 μm^2^ s^–1^) and comparable to Au–TiO_2_ micromotors under UV light irradiation (0.4 μm^2^ s^–1^). This trend was discovered also for velocity values (0.6 and 2.8 μm s^–1^ for Au–TiO_2_ micromotors in dark and light conditions, 3.3 and 5.5 μm s^–1^ for Ag–TiO_2_ micromotors in dark and light conditions). For both micromotors, the velocity under UV light irradiation in 0.1 wt % H_2_O_2_ improved compared to pure water. In this regard, particularly relevant is the enhancement of the velocity of Ag–TiO_2_ micromotors.

The most remarkable finding of motion experiments in 0.1 wt % H_2_O_2_ is that the presence of the fuel reverts the paradigm of the higher built-in electric field at the metal–semiconductor interface. Indeed, it was revealed that the catalytic properties of the metal may exceed the constrictions deriving from the energy band bending of the metal–semiconductor Schottky junction, as it occurred for Ag–TiO_2_ micromotors in the presence of 0.1 wt % H_2_O_2_. On these bases, the behavior of the two types of micromotors was described according to the scheme illustrated in Figure 4d. In the dark, both Au and Ag metal layers decomposed the H_2_O_2_ fuel based on the following reaction.
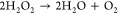
6However, the superior catalytic properties of Ag compared to Au concerning H_2_O_2_ decomposition led to the generation of a larger product concentration gradient, which allowed overcoming Brownian motion and achieving the self-propulsion via the self-diffusiophoretic mechanism. As a consequence, under UV light irradiation, Au–TiO_2_ micromotors marginally benefited from the presence of H_2_O_2_ and moved with a velocity slightly higher than that of pure water. On the contrary, the synergy between Ag catalytic activity and self-electrophoresis let Ag–TiO_2_ micromotors reach the highest velocity despite the lower built-in electric field of the Ag–TiO_2_ contact.

Even though Ag–TiO_2_ micromotors exhibited a more powerful self-propulsion, it is generally reported that the Ag layer easily dissolves during the catalytic reaction with an H_2_O_2_ solution. Therefore, for practical applications, it is crucial to evaluate the potential release of Ag^+^ ions in water. For this purpose, the Ag–TiO_2_ micromotors were immersed in 0.1 wt % H_2_O_2_ under UV light irradiation for 2 h. At the end of the experiment, the solution was analyzed by inductively coupled plasma-mass spectrometry (ICP-MS), which revealed a concentration of Ag^+^ ions of 0.18 mg L^–1^. Although this value is slightly above the secondary maximum contaminant limit (SMCL) of 0.1 mg L^–1^ set by the United States Environmental Protection Agency (U.S. EPA) and the World Health Organization (WHO),^53^ it can be decreased by reducing the concentration of the micromotors. On the other hand, the ability of the Ag–TiO_2_ micromotors to release a large number of Ag^+^ ions during their self-propulsion in an H_2_O_2_ solution can be beneficial for specific applications, such as the elimination of bacteria and the eradication of bacterial biofilms, due to Ag^+^ ions antibacterial properties.^54,55^

### Polymer Degradation Application

**Figure 5:**
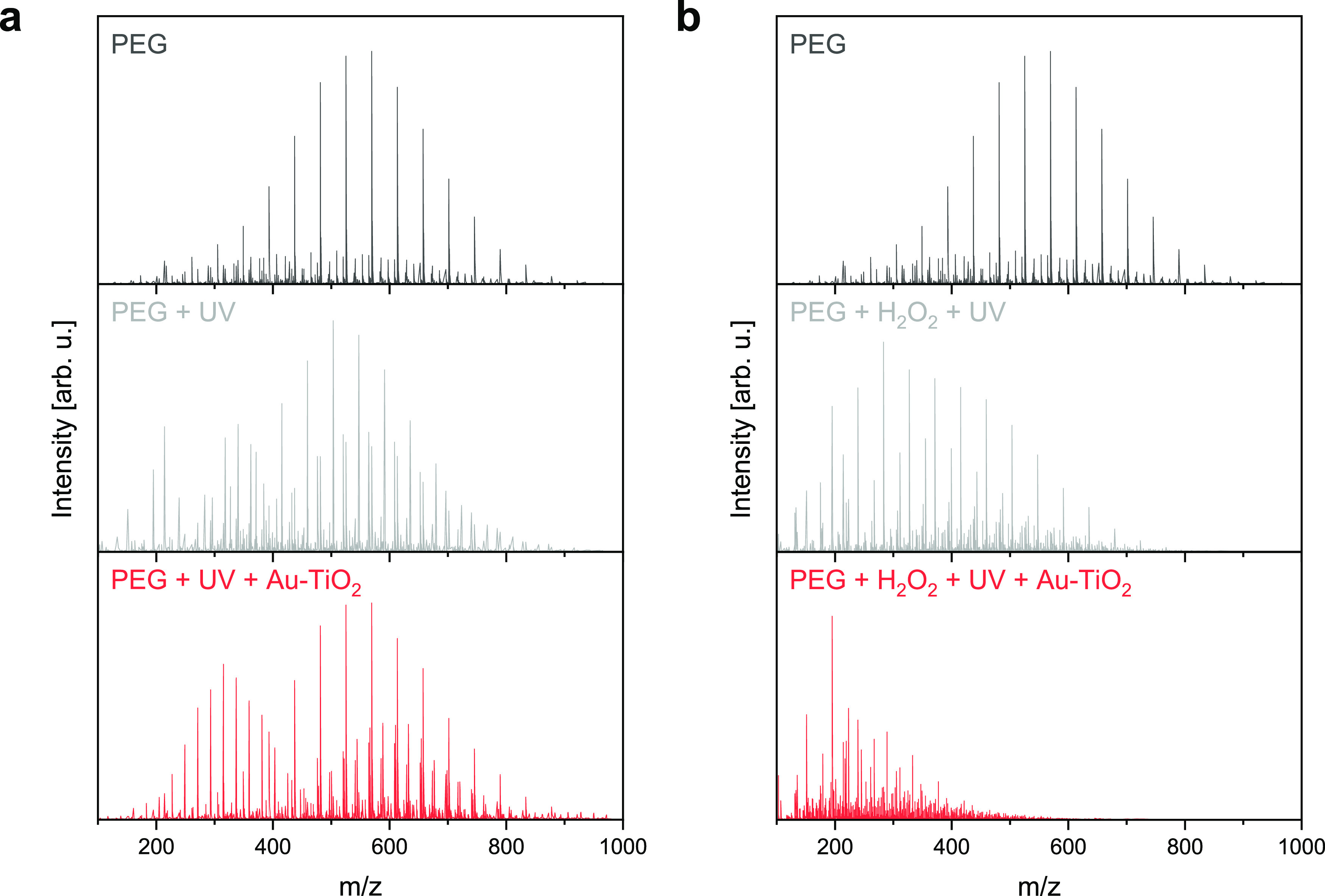
Polymer degradation experiments.(a) From top to bottom: ESI-MS spectra of untreated PEG, PEG treated with UV-light irradiation in pure water for 8 h, and PEG treated with Au–TiO_2_ micromotors under UV light irradiation in pure water for 8 h. (b) From top to bottom: ESI-MS spectra of untreated PEG, PEG treated with UV-light irradiation in 0.1 wt % H_2_O_2_ for 8 h, and PEG treated with Au–TiO_2_ micromotors under UV light irradiation in 0.1 wt % H_2_O_2_ for 8 h.

In a previous study, similar MXene-derived γ-Fe_2_O_3_/Pt/TiO_2_ microrobots were applied to preconcentrate and detect nanoplastics in water via tunable electrostatic interactions and electrochemical measurements using miniaturized electrodes.^34^ Conversely, in this study, MXene-derived metal–TiO_2_ micromotors were applied for the degradation of synthetic PEG chains with a molecular weight of ∼600 g mol^–1^. This polymer is widely used in cosmetics and pharmaceutical formulations. It was selected as a model for persistent water pollutants since its synthetic nature and the covalent bonds linking its organic subunits (monomers) make degradation challenging.^56,57^ In addition, PEG degradation process can be accurately monitored by mass spectrometry techniques revealing the presence of oligomers even in nanomole concentration.^58^ PEG degradation experiments were initially performed under UV light irradiation in pure water. Consequently, Au–TiO_2_ micromotors were selected as the optimal micromotors for these experiments due to their higher velocity under this condition. PEG degradation was evaluated by electrospray ionization mass spectrometry (ESI-MS). Figure 5a compares the spectra of PEG in pure water before any treatment, PEG treated with UV light irradiation in pure water for 8 h, and PEG treated with Au–TiO_2_ micromotors under UV light irradiation in pure water for 8 h. The mass distribution of untreated PEG was centered around 600 *m*/*z*, as expected. After the treatment with UV light irradiation, the measured mass distribution slightly shifted to lower *m*/*z* values and new signals appeared in the *m*/*z* region 100–300, indicating that the prolonged exposure to UV light initiated the degradation of the polymer. After the treatment with Au–TiO_2_ micromotors under UV light irradiation, the mass distribution further shifted to lower *m*/*z* values, while a second and pronounced mass distribution, centered around 300 *m*/*z*, suggested a higher PEG oxidation. This is due to the photocatalytic activity of Au–TiO_2_ micromotors, which produced reactive oxygen species (ROS) during their self-propulsion, breaking and oxidizing the PEG chains as revealed from mass peak assessment.

In pure water, the polymer was not thoroughly degraded. Therefore, H_2_O_2_ was involved in the treatments to improve the degradation efficiency of PEG. Of note, H_2_O_2_ toxicity, even at concentrations as low as 0.1 wt %, limits its applicability, especially in the biomedical field. However, in water purification applications, H_2_O_2_ is often used in combination with (photo)catalysts and light irradiation, allowing Fenton and photo-Fenton reactions that enhance the production of ROS and accelerate the degradation process. On these bases, the experiments were repeated in the presence of 0.1 wt % H_2_O_2_ and the obtained spectra are reported in Figure 5b. A remarkable degradation of PEG was obtained using H_2_O_2_ and UV light irradiation due to the UV light breaking the H_2_O_2_ molecule to form hydroxyl radicals (OH^·^). In the presence of the Au–TiO_2_ micromotors, the mass spectrum presented almost no signal for *m*/*z* above 400, and a narrow mass distribution around *m*/*z* 200 suggested that the long PEG chains were broken into pieces with lower molecular weights.

In principle, the degradation efficiency can be further improved by increasing the amount of photocatalysts, H_2_O_2_ concentration, UV light irradiation intensity, or treatment duration. In this regard, by prolonging the treatment with Au–TiO_2_ micromotors, 0.1 wt % H_2_O_2,_ and UV light irradiation from 8 to 16 h, a superior PEG degradation was obtained (Figure S5).

Control experiments were also performed to compare the MXene-derived Au–TiO_2_ micromotors with Au–TiO_2_ microparticles prepared by the sputtering deposition of an Au layer on purchased TiO_2_ microparticles. In particular, PEG was treated with MXene-derived or commercial Au–TiO_2_ microparticles under UV light irradiation in pure water and 0.1 wt % H_2_O_2_ for 8 h. The acquired mass spectra are compared in Figure S6. In pure water, the performances of the MXene-derived Au–TiO_2_ micromotors and commercial Au–TiO_2_ micromotors were comparable, i.e., the distribution of the degradation products was similar for the two cases. Instead, in the presence of H_2_O_2_, the MXene-derived Au–TiO_2_ micromotors showed a significant improvement compared with their cheaper counterpart. This improvement is evident since the mass spectra of the latter still presented distinct signals in the *m*/*z* region 400–700 and may be attributed to the larger exposed surface of multilayered TiO_2_ microparticles. Despite demonstrating slightly inferior performance in PEG degradation compared to MXene-derived TiO_2_, commercial TiO_2_ proved more advantageous overall due to its lower cost. Indeed, the preparation of MXene-derived TiO_2_ involves more expensive precursors and additional preparation steps.

## Conclusions

This study investigated the light-powered motion of metal–semiconductor micromotors based on MXene-derived TiO_2_ microparticles in contact with metals (Au and Ag) characterized by different work functions, leading to diverse electronic properties for the metal–TiO_2_ interface. The fabrication involved transforming exfoliated Ti_3_C_2_T_*x*_ MXene microparticles into multilayered TiO_2_ by thermal annealing and then depositing thin Au or Ag layers on their surface, asymmetrically, by the sputtering technique. The motion behavior of the resulting MXene-derived metal–TiO_2_ micromotors was studied in pure water and 0.1 wt % H_2_O_2_ as a fuel. Under UV light irradiation, both types of micromotors showed self-propulsion in pure water via self-electrophoresis, with Au–TiO_2_ micromotors exhibiting velocities higher than those of Ag–TiO_2_ micromotors. This finding was explained by the more intense built-in electric field at the Au–TiO_2_ Schottky junction, which improves the photogenerated electron–hole pairs separation in TiO_2_ and hole accumulation at the interface, as indicated by numerical simulations, which contribute to the self-electrophoretic motion mechanism. In the presence of H_2_O_2_, the behavior changed significantly. Au–TiO_2_ micromotors displayed marginal improvement in velocity under exposure to UV light, while Ag–TiO_2_ micromotors manifested autonomous motion even in the absence of UV light by self-diffusiophoresis and the highest velocity under UV light irradiation due to the synergy between Ag’s catalytic activity and self-electrophoresis. Overall, the study demonstrates the importance of metal–semiconductor interfaces and the competition with metal catalytic properties in the light-driven motion of micro- and nanomotors, highlighting the impact of the metal choice on their performance. In addition, the developed Au–TiO_2_ micromotors proved a great potential in the remediation of polymer-contaminated water, cleaving PEG chains by photocatalysis in pure water and photo-Fenton reaction in the presence of H_2_O_2_. These conclusions provide valuable insights into designing and optimizing metal–semiconductor interfaces for photocatalytic micro- and nanomotors and their applications. Furthermore, it is anticipated that the absence of potential barriers obstructing the flow of electrons and the built-in electric field pointing to the semiconductor in Ohmic junctions can favor photogenerated charge separation within the semiconductor and, at the same time, successive electron transfer to the metal, resulting in enhanced self-propulsion. To verify this hypothesis, future investigations will focus on comparing the performance of metal–semiconductor Ohmic and Schottky junctions in the light-powered micro- and nanomotors field.

## Experimental Section

### Fabrication of MXene-Derived Metal–TiO_2_ Micromotors

Exfoliated Ti_3_C_2_T_*x*_ MXene microparticles (XFNano, China) were suspended in pure water (18 MΩ cm) at a concentration of 1 mg mL^–1^ and sonicated for 1 h in a bath sonicator. Then, the suspension was dropped on microscope glass slides, serving as a substrate, and dried overnight. The resulting Ti_3_C_2_T_*x*_ MXene films were transferred into a tubular furnace and underwent a thermal annealing process in synthetic air (2.5 L min^–1^) at 550 °C for 0 min, i.e., the temperature rump-up immediately followed by the temperature ramp-down, with a heating rate of 10 °C min^–1^, obtaining photocatalytic MXene-derived TiO_2_ microparticles.

Afterward, highly asymmetric Janus structures were obtained by depositing thin metal layers on the annealed microparticles by a sputtering technique. In particular, an Emitech K550X sputter coater (Quorum, Ringmer, East Sussex, U.K.) was used to deposit Au and Ag layers from high purity targets (99%) under the sputtering conditions of 50 mA current and 12 min deposition time, fabricating MXene-derived Au–TiO_2_ and Ag–TiO_2_ micromotors with a nominal metal layer thickness of about 80 nm. Finally, a scalpel was used to detach the micromotors from the substrates mechanically.

### Characterization Techniques

Surface morphology and elemental composition of samples were characterized by a Gemini field emission SEM Carl Zeiss Supra 25. Raman spectra of samples before and after the thermal annealing process were acquired in backscattering geometry using a HORIBA Jobin-Yvon system coupled to an Olympus BX41 microscope. He–Ne laser radiation (633 nm wavelength, ∼5 mW power) was focused to a spot size of 1 μm through a 100× microscope objective. A 550 mm focal length spectrometer with 1800 lines mm^–1^ grating was used to collect the Raman emission from samples. The optical bandgap of MXene-derived TiO_2_ microparticles was determined using a PerkinElmer LAMBDA 1050+ UV/vis/NIR spectrophotometer furnished with an integrating sphere.

### Motion Experiments

The light-powered motion of MXene-derived micromotors was tested in pure water and 0.1 wt % H_2_O_2_ (Merck, 30 wt %) without any surfactant using a Leica DMI4000 B inverted optical microscope equipped with a Basler digital camera (acA1920-155uc). A light source (Leica EL6000), coupled with fluorescence filter cubes, allowed micromotors to be irradiated with UV light (375 nm wavelength, ∼50 mW cm^–2^ intensity) or visible light (480 nm wavelength, ∼200 mW cm^–2^ intensity) to induce their movement. A control experiment was also performed on a raw wastewater sample.

Movies of micromotors’ motion behavior were recorded at a frame rate of 10 fps through Pylon Viewer software and analyzed using Fiji software to obtain their trajectories and calculate their MSD and velocity.

### Ag Dissolution Experiment

To investigate the potential corrosion of the Ag layer of MXene-derived Ag–TiO_2_ micromotors in the presence of H_2_O_2_, the micromotors (1 mg mL^–1^) were left in 0.1% H_2_O_2_ under UV light irradiation for 2 h. Then, the suspension was centrifuged at 4000 rpm for 5 min to separate the micromotors from the supernatant, which was further analyzed using a Nexion 300X ICP/MS instrument (PerkinElmer Inc., Waltham, Massachusetts, USA) using the kinetic energy discrimination mode (KED) for interference suppression. Before analysis, the sampled solution was diluted, acidified with nitric acid, and added to the internal standards required for quantifying Ag^+^ ions. The instrumental analyses were repeated 3 times for higher accuracy and were validated by comparing it with a standard reference material, SRM 1643f Trace Elements in Water.

### Polymer Degradation Experiments

PEG degradation experiments were performed in UV light-transparent cuvettes containing aqueous suspensions with 1 mg mL^–1^ synthetic PEG 600, 1 mg mL^–1^ MXene-derived Au–TiO_2_ micromotors, and, eventually, 0.1 wt % H_2_O_2_. The cuvettes were exposed to UV light irradiation for different durations (8 or 16 h) using a 365 nm UV LED lamp. At the end of the treatment, the suspensions were centrifugated at 4000 rpm for 5 min to separate the micromotors from the supernatants, which were further analyzed to record mass spectra by ESI-MS. Control experiments were performed under the same experimental conditions with Au–TiO_2_ microparticles, prepared by the sputtering deposition of an Au layer on purchased TiO_2_ microparticles (Merck, SKU 224227). ESI-MS was performed using a Thermo Scientific Orbitrap Exploris 120 (Thermo Fisher Scientific, Bremen, Germany) with a heated electrospray ionization interface. Mass spectra were recorded in positive ion mode, by direct infusion of sample solutions, in the *m*/*z* range 100–1000 at a resolving power of 60000 (full-width-at-half-maximum, RFWHM, at *m*/*z* 200), under the following conditions: capillary temperature 300 °C, capillary voltage 3.5 kV, nebulizer gas (nitrogen) flow rate of 10 arbitrary units, and auxiliary gas flow rate of 2 arbitrary units. The Orbitrap MS system was tuned and calibrated using a Thermo Scientific Pierce^TM^ FlexMix^TM^ calibration solution. Data acquisition and analysis were performed using the Excalibur software.

## Supporting Information Available

Simulated electron concentration at the metal–TiO_2_ interface; SEM image of a Ti_3_C_2_T_*x*_ MXene microparticle; trajectories of MXene-derived TiO_2_ microparticles under UV light irradiation in pure water; MSD data fitting results; photograph and micrograph of the raw wastewater sample; ESI-MS spectra of PEG treated for different durations; and ESI-MS spectra of PEG treated with MXene-derived and commercial Au–TiO_2_ (PDF)
MXene-derived micromotors under successive on–off switching of UV light irradiation in pure water (AVI)
MXene-derived micromotors under successive on–off switching of UV light irradiation in 0.1 wt % H_2_O_2_ (AVI)
MXene-derived micromotors under UV light irradiation in raw wastewater (AVI)
MXene-derived micromotors under visible light irradiation in pure water (AVI)

## Supplementary Material

am3c13470_si_001.pdf

am3c13470_si_002.avi

am3c13470_si_003.avi

am3c13470_si_004.avi

am3c13470_si_005.avi
