# Supplementary material for: Band Engineering versus Catalysis: Enhancing the Self-Propulsion of Light-Powered MXene-Derived Metal–TiO2 Micromotors To Degrade Polymer Chains
Source: ACS Appl Mater Interfaces. 2023 Dec 22;16(1):1293–307. doi: 10.1021/acsami.3c13470 (PMC10788834; doi:10.1021/acsami.3c13470)
Supplement: Supplementary file 1 — am3c13470_si_001.pdf [file am3c13470_si_001.pdf]

Supporting Information

Band Engineering versus Catalysis: Enhancing the Self-Propulsion of Light-Powered MXene-Derived Metal–TiO_2 Micromotors To Degrade Polymer Chains

AUTHOR NAMES

Mario Urso[1,2*], Luca Bruno[1,2], Sandro Dattilo[3], Sabrina C. Carroccio[3], Salvo Mirabella[1,2]

AUTHOR ADDRESS

[1] Dipartimento di Fisica e Astronomia "Ettore Majorana", Università degli Studi di Catania, via S. Sofia 64, 95123 Catania, Italy

[2] CNR-IMM, via S. Sofia 64, 95123 Catania, Italy

[3] CNR-IPCB, Catania Unit, via Paolo Gaifami 18, 95126 Catania, Italy

ORCID

Mario Urso: https://orcid.org/0000-0001-7993-8138

Luca Bruno: https://orcid.org/0000-0003-2710-1154

Sandro Dattilo: https: https://orcid.org/0000-0002-3127-9580

Sabrina Carola Carroccio: https://orcid.org/0000-0002-9645-0369

Salvo Mirabella: https://orcid.org/0000-0002-9559-4862

E-mail: mario.urso@dfa.unict.it

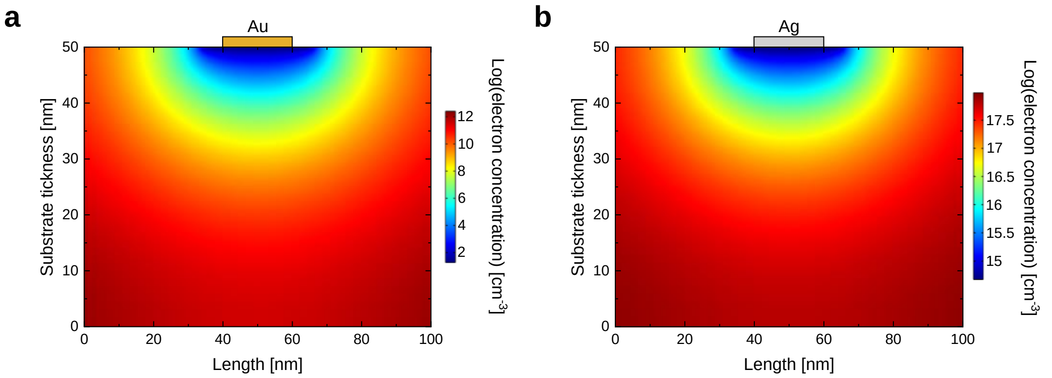

Figure S1. Simulated electron concentration at the metal–TiO_2 interface for **a** Au–TiO_2 and **b** Ag–TiO_2 junctions formed by a metal nanoparticle (20 nm in diameter) on the surface of a TiO_2 microparticle.

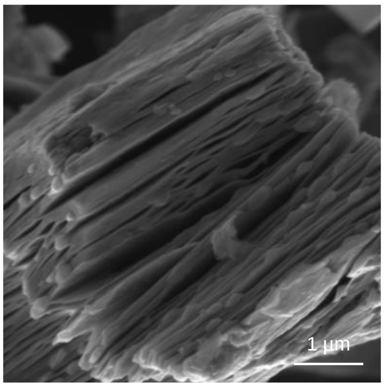

Figure S2. SEM image of a $Ti_3C_2T_x$ MXene microparticle.

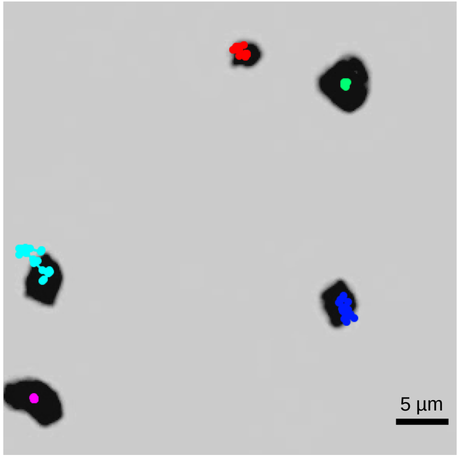

Figure S3. Trajectories of MXene-derived TiO_2 microparticles under UV light irradiation in pure water for 20 s.

Table S1. Mean squared displacement (MSD) data fitting results based on Eq. (3) and (4) in the manuscript.

Sample	H_2O_2 [wt %]	Irradiation condition	Fit	R^2	D [$\mu m^2\ s^{-1}$]	v [$\mu m\ s^{-1}$]
Au–TiO_2	0	Dark	Linear	0.994	0.0273 ± 0.0005	
		Light	Parabola	0.999	0.08 ± 0.03	2.6 ± 0.3
	0.1	Dark	Parabola	0.995	0.045 ± 0.005	0.6 ± 0.1
		Light	Parabola	0.999	0.4 ± 0.03	2.8 ± 0.4
Ag–TiO_2	0	Dark	Linear	0.990	0.029 ± 0.001	
		Light	Parabola	0.998	0.10 ± 0.03	2.1 ± 0.3
	0.1	Dark	Parabola	0.999	0.33 ± 0.05	3.3 ± 0.4
		Light	Parabola	0.999	0.6 ± 0.1	5.5 ± 0.7

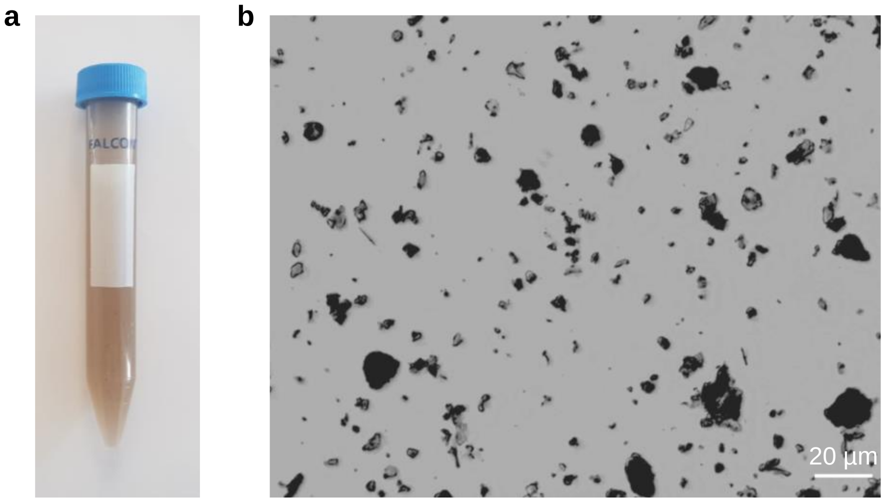

Figure S4. a Photograph and **b** micrograph of the raw wastewater sample used for the motion experiment of Au–TiO_2 and Ag–TiO_2 micromotors under UV light irradiation.

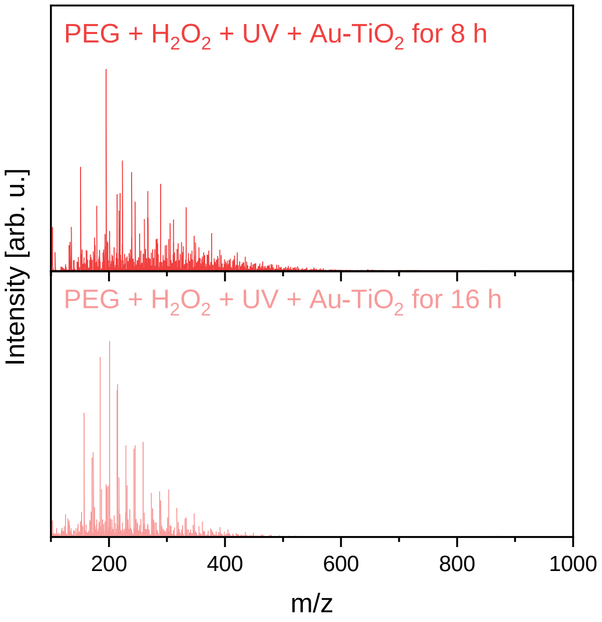

Figure S5. From top to bottom, ESI-MS spectra of PEG treated with Au–TiO_2 micromotors under UV light irradiation in 0.1 wt % H_2O_2 for 8 h and 16 h.

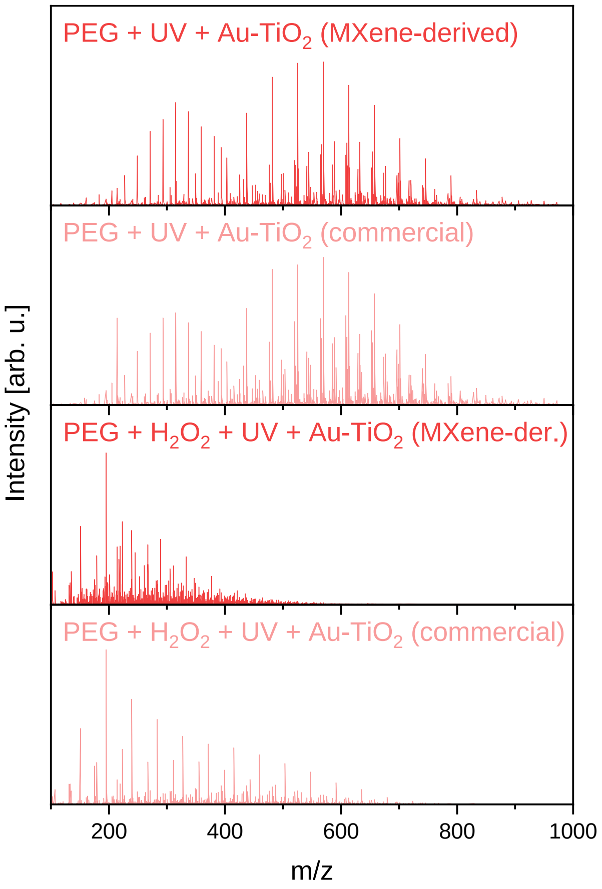

Figure S6. ESI-MS spectra of PEG treated with Au–TiO_2 micromotors fabricated from MXene-derived TiO_2 microparticles and commercial TiO_2 microparticles under UV light irradiation in pure water and 0.1 wt % H_2O_2 for 8 h.
